# Peri-conceptional or pregnancy exposure of HPV vaccination and the risk of spontaneous abortion: a systematic review and meta-analysis

**DOI:** 10.1186/s12884-019-2425-1

**Published:** 2019-08-19

**Authors:** Jing Tan, Yi-quan Xiong, Qiao He, Yan-mei Liu, Wen Wang, Meng Chen, Kang Zou, Xing-hui Liu, Xin Sun

**Affiliations:** 1 Chinese Evidence-based Medicine Centre and CREAT Group, West China Hospital, Sichuan University, Chengdu, 610041 Sichuan China; 2 Department of Obstetrics and Gynecology, West China Second University Hospital, Sichuan University, Chengdu, 610041 Sichuan China

**Keywords:** Human papillomavirus vaccine, Pregnancy, Spontaneous abortion, Meta-analysis

## Abstract

**Background:**

To assess whether the peri-conceptional or pregnancy exposure of human papillomavirus (HPV) vaccination would increase the risk of spontaneous abortion.

**Methods:**

We searched PubMed, Embase, and Cochrane Central Register of Controlled Trials for clinical trials and observational studies that investigated the association between exposure of HPV vaccines (2vHPV, 4vHPV or 9vHPV) during peri-conceptional period or pregnancy and spontaneous abortion before 28 gestational weeks. We pooled data from 2vHPV, 4vHPV and 9vHPV separately. Subgroup analyses were conducted according to data sources, and raw data or adjusted data.

**Results:**

Seven observational studies were eligible and all studies were low risk of bias. Meta-analyses suggested that 2vHPV vaccination did not increase the risk of spontaneous abortion regardless of exposure period during 90 days before last menstrual period (LMP) or pregnancy: risk ratio, 95% confidence intervals (RR, 95% CI), 1.15 (0.95–1.39), and 45 days before LMP or pregnancy: 1.28 (0.96–1.70). However, 2vHPV vaccination during Pre-45 days to LMP seemed to increase the risk of spontaneous abortion: 1.59 (1.04–2.45). The current evidence did not support the association between 4vHPV vaccination and spontaneous abortion regardless of exposure period during 45 days before LMP or pregnancy: 0.88 (0.73–1.06); and 45 days before LMP: 1.00 (0.80–1.24). Additionally, 9vHPV during within 30 days of conception also seemed to increase the risk: 2.04 (1.28–3.24).

**Conclusions:**

The association between peri-conceptional or pregnancy exposure of HPV vaccine and spontaneous abortion is still uncertain, and additional research is warranted to assess the impact of exposure of HPV vaccination on spontaneous abortion.

**Electronic supplementary material:**

The online version of this article (10.1186/s12884-019-2425-1) contains supplementary material, which is available to authorized users.

## Introduction

Since licensed by the U.S. Food and Drug Administration in 2006, human papillomavirus (HPV) vaccine, has been used among 72 million females worldwide [1, 2]. Three HPV vaccines, including bivalent vaccine (2vHPV), quadrivalent vaccine (4vHPV) and nonavalent vaccine (9vHPV), are currently available [3]. In many countries, HPV vaccines are recommended as part of the routine vaccination for female children aged 11–13 years [1, 4]. Those females younger than 25 or 26 years, who have not received vaccination, are also recommended [5, 6]. As a result, a large number of women at childbearing age may be exposed to HPV vaccination. These include those who may be inadvertently vaccinated during peri-conceptional period or pregnancy, particularly those who were unplanned or unrecognized pregnant [7].

Although all the three HPV vaccines are inactivated, given the absence of well controlled studies in pregnant women, pregnancy is listed as a precaution condition for HPV vaccination [4, 8]. Concerns have arisen as to whether the exposure of HPV vaccines before or during pregnancy would increase the risk of serious adverse pregnancy outcomes, such as spontaneous abortion, congenital defect, premature birth and stillbirth [9–11]. Several studies have investigated the association between HPV vaccination and risk of adverse pregnancy outcomes [2, 12–20]. In 2010, Wacholder et al. found higher risk of spontaneous abortion among women who conceived during less than 90 days from 2vHPV vaccination than among those in the control group (13.7% vs. 9.2%) [17]. In another analysis, the authors showed that the incidence of spontaneous abortion among women who conceived within 30 days before or after 9vHPV vaccination was higher than that of conceived not within this specific period (17.5% vs. 8.6%) [13].

Up to now, it remains largely uncertain whether the unintended exposure of HPV vaccination at the peri-conceptional period or during pregnancy will increase the risk of spontaneous abortion. This represents an important knowledge gap. Therefore, we conducted a systematic review and meta-analysis of all relevant clinical research evidence to address this unanswered important clinical question. In particular, we examined all of the available HPV vaccines to ensure a balanced assessment.

## Methods

This study was conducted and reported according to the Preferred Reporting Items for Systematic Reviews and Meta-Analyses (PRISMA) guideline [21], with review protocol prospectively registered in PROSPERO (CRD42019120198).

### Eligibility criteria

We included clinical trials, or cohort studies if they assessed the association between peri-conceptional or pregnancy exposure of HPV vaccination and the risk of spontaneous abortion; reported usable outcome data (e.g. relative risk or the associated events); reported the time interval between vaccination and conception. When there were multiple publications for a same study, we used the data from the most recent or comprehensive ones.

### Literature search

PubMed, Embase, and Cochrane Central Register of Controlled Trials (CENTRAL) were searched from the inception to 23nd July 2018 with updating on 9th April, 2019. We combined both Medical Subject Headings (MeSH) and free text terms for identifying relevant articles (see detailed search strategy in Additional file 1). Reference lists of included articles were also checked for additional relevant publications.

### Study process

Two reviewers independently screened the title, abstract, and full text according to the above inclusion criteria. Reviewers resolved disagreement through discussion or, if required, adjudication by a third reviewer. A standardized and pilot-tested form was used to extract data from each eligible study, including study characteristics (e.g., first author name, year of publication, country of origin, study design, and sample size), details in exposure group and control group (e.g., type of HPV vaccine, exposure time of HPV vaccine, type of control), and outcomes (e.g., number of events and patients included for analyses in each group). For observational studies, we also collected methods used to control confounding, and reported adjustment factors if available.

### Risk of bias assessment

We assessed the risk of bias of cohort studies by using the Newcastle–Ottawa Scale (NOS) [22, 23]. In this scale, studies are scored across three categories: selection of subjects, comparability of study groups, and the assessment of exposure. Studies were graded on an ordinal scoring scale with a maximum score of 9. Higher score represents higher quality of study.

### Statistical analysis

**Fig. 1:**
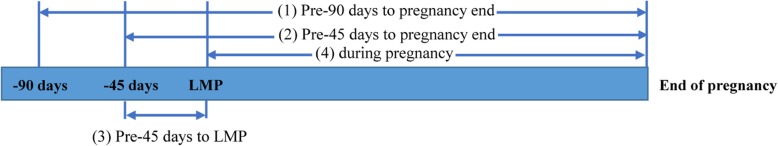
Exposure windows of HPV vaccination during peri-conceptional period or pregnancy. LMP, last menstrual period

We analyzed the three types of HPV vaccines, 2vHPV, 4vHPV or 9vHPV, separately. Exposure time of HPV vaccination during peri-conception or pregnancy was generalized as four exposure windows in this study (Fig. 1): (1) Pre-90 days to pregnancy end, vaccination within 90 days before last menstrual period (LMP) and any time during pregnancy; (2) Pre-45 days to pregnancy end, vaccination within 45 days before LMP and any time during pregnancy; (3) Pre-45 days to LMP, vaccination within 45 days before LMP; (4) during pregnancy, vaccination within first 22 gestation weeks.

Summary measures were reported as relative risk (RR) and risk difference (RD) with 95% confidence interval (CI). Between-study heterogeneity was estimated by the clinical features of included studies and the Cochran chi-square test and *I*^*2*^ statistic [24], and significant heterogeneity was defined as *I*^*2*^ ≥ 50%. Pooled results were calculated with a fixed effects model when heterogeneity was not significant (*I*^*2*^ < 50%); otherwise, a random-effects model was applied. We pre-specified the following subgroup hypotheses for exploring heterogeneity (1) different sources of data (clinical trials vs. databases) in original studies; and (2) different type of data (raw data vs. adjusted data). Sensitivity analyses were conducted to explore the robustness of our findings using different effect measures (when original effect size was RR, OR was used to instead of RR), and statistical models (when original statistical model was fixed-effects model, random-effects model was used to instead of fixed-effects model). We were unable to examine the publication bias due to small number of studies [25].

## Results

### Characteristics of the studies included in this meta-analysis

**Fig. 2:**
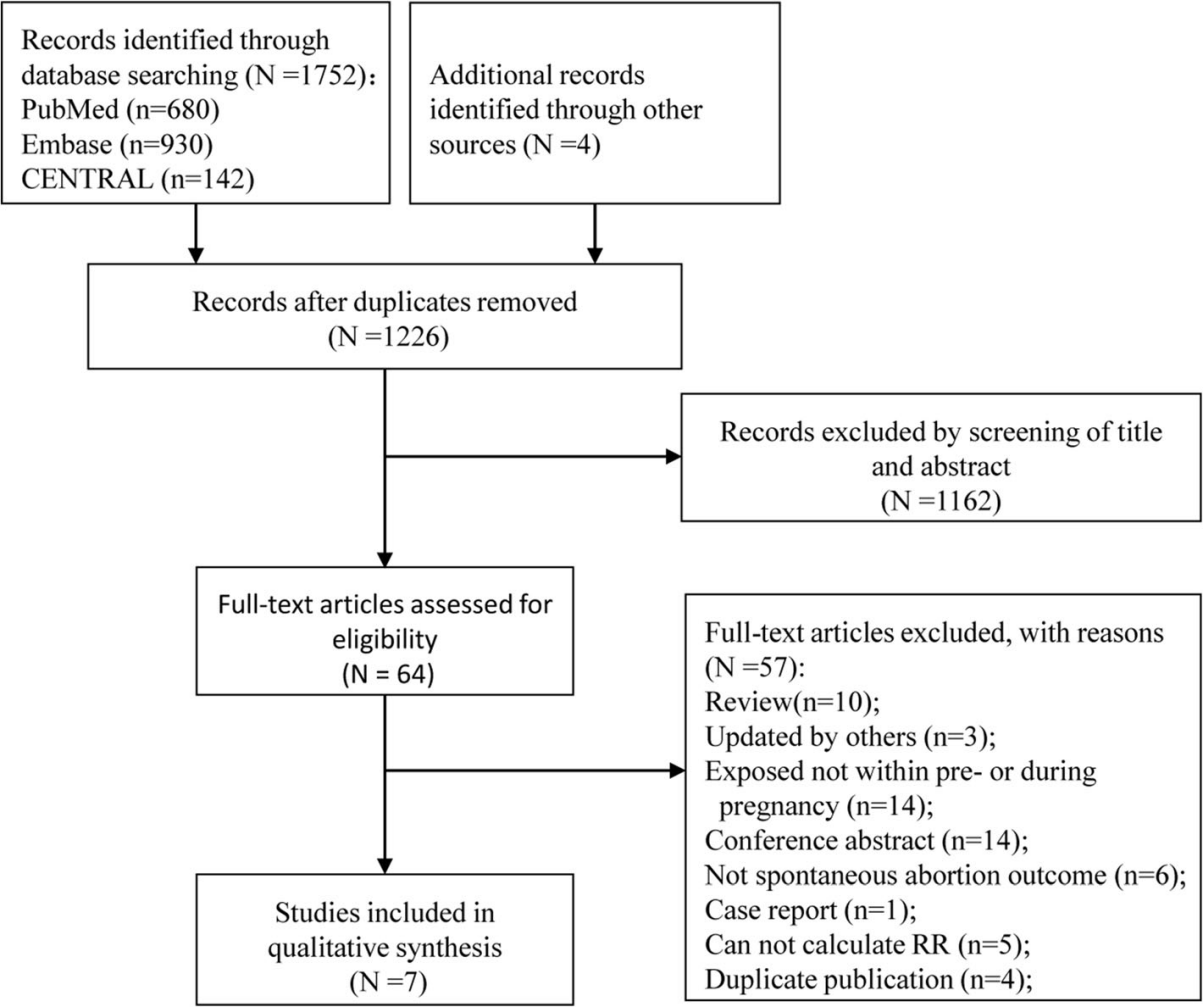
Flow diagram of the studies identified in the meta-analysis. CENTRAL, Cochrane Central Register of Controlled Trials; RR, relative risk; RD, risk difference

The systematic literature search identified 1752 articles. After abstract and full text screening, seven studies were included in this study [2, 12–16, 18]. The selection process was shown in Fig. 2. The main characteristics of included studies were summarized in Table 1. There were three studies [14–16], four studies [2, 12, 13, 18] and one study [13], focused on the effect of 2vHPV, 4vHPV, and 9vHPV vaccine, respectively. In four studies [2, 12, 13, 15], pregnant women in control group received HPV vaccination in specific time period, which was not overlap with the exposure windows in exposure group and was farther away from conception. In another three studies [14, 16, 18], pregnant women in control group did not receive HPV vaccination. There are four observational studies, included: Kharbanda et al. [12] was conducted within the data from Vaccine Safety Datalink of seven sites in USA between January 2008 and November 2014; Scheller et al. [2] used nationwide registers to identify the women who had vaccine exposure in Denmark between October 2006 and November 2013; Baril et al. [15] included women registered with the Clinical Practice Research Datalink General Practice OnLine Database in the United Kingdom, who received at least one 2vHPV dose between September 2008 and June 2011; and Panagiotou et al. [14] was a long term follow-up of a randomized, double blinded trial combined with an independent unvaccinated population based cohort in Costa Rica. The other three studies reported combined results of more than one trial, including combined analysis of forty-two (conducted in 40 countries) [16], seven (conducted in 31 countries) [13], and five trials (conducted in multiple countries) [18]. According to NOS scale, all included studies were of high quality.

**Table 1:** Basic characteristics of the studies included in this meta-analysis

First Author	Study design	Study time	Country	Exposure vaccine	Vaccination exposure time of HPV vaccine group	Comparison	Vaccination (or placebo) exposure time of control group
Kharbanda (2018) [12]	retrospective observational cohort	2008.01–2014.11	the United States	4vHPV	within (1) peripregnancy: 42 days before LMP; (2) during pregnancy: first 19 weeks of pregnancy; (3) peri or during pregnancy: 42 days before LMP to 19 weeks of gestation	4vHPV	4vHPV vaccinated within 16 to 22 weeks before LMP
Scheller (2017) [2]	retrospective observational cohort	2006.10.1–2013.11	Denmark	4vHPV	within 7 to 22 weeks of pregnancy	4vHPV	4vHPV not vaccinated during 7 to 22 weeks of pregnancy
Moreira (2016) [13]	combined analysis of 7 phase three clinical trials	NA	31 countries	9vHPV and 4vHPV	within 30 days before and after conception ^a^	9vHPV and 4vHPV	9vHPV or 4vHPV not vaccinated during 30 days before and after conception
Panagiotou (2015) [14]	long term observational follow-up in the Costa Rica HPV Vaccine Trial	2004.6–2013.12	Costa Rica	2vHPV	within 90 days before and after conception	hepatitis A vaccine or unvaccination	not vaccinated HPV vaccine
Baril (2015) [15]	retrospective observational cohort	2008.9–2011.6	United Kingdom	2vHPV	within 90 days before and 30 days after LMP	2vHPV	2vHPV vaccinated within 4 to 18 months before LMP
Angelo (2014) [16]	pooled analysis of data from 42 completed/ongoing clinical studies	NA	40 countries	2vHPV	within (1) 45 days before and 30 days after pregnancy; (2) 60 days before pregnancy to pregnancy end; (3) first 12 weeks of pregnancy	placebo (Al (OH)_3_), Havrix, hepatitis A vaccine, Gardasil, Menactra, Boostrix, Boostrix Polio, Engerix B or Twinrix Paediatri	vaccination with control vaccine within 60 days before pregnancy to pregnancy end
Garland (2009) [18]	combined analysis of five randomized controlled trials	NA	multiple countries	4vHPV	within 30 days before conception	placebo	vaccination with placebo within 30 days before conception

^a^conception date was calculated as date of last menstrual period plus 14 days; *2vHPV* bivalent HPV vaccine, *4vHPV* quadrivalent HPV vaccine, *9vHPV* nonavalent HPV vaccine, *GW* Gestational weeks, *LMP* Last menstrual period, *NA* Not available

### Association between 2vHPV vaccination and spontaneous abortion

**Fig. 3:**
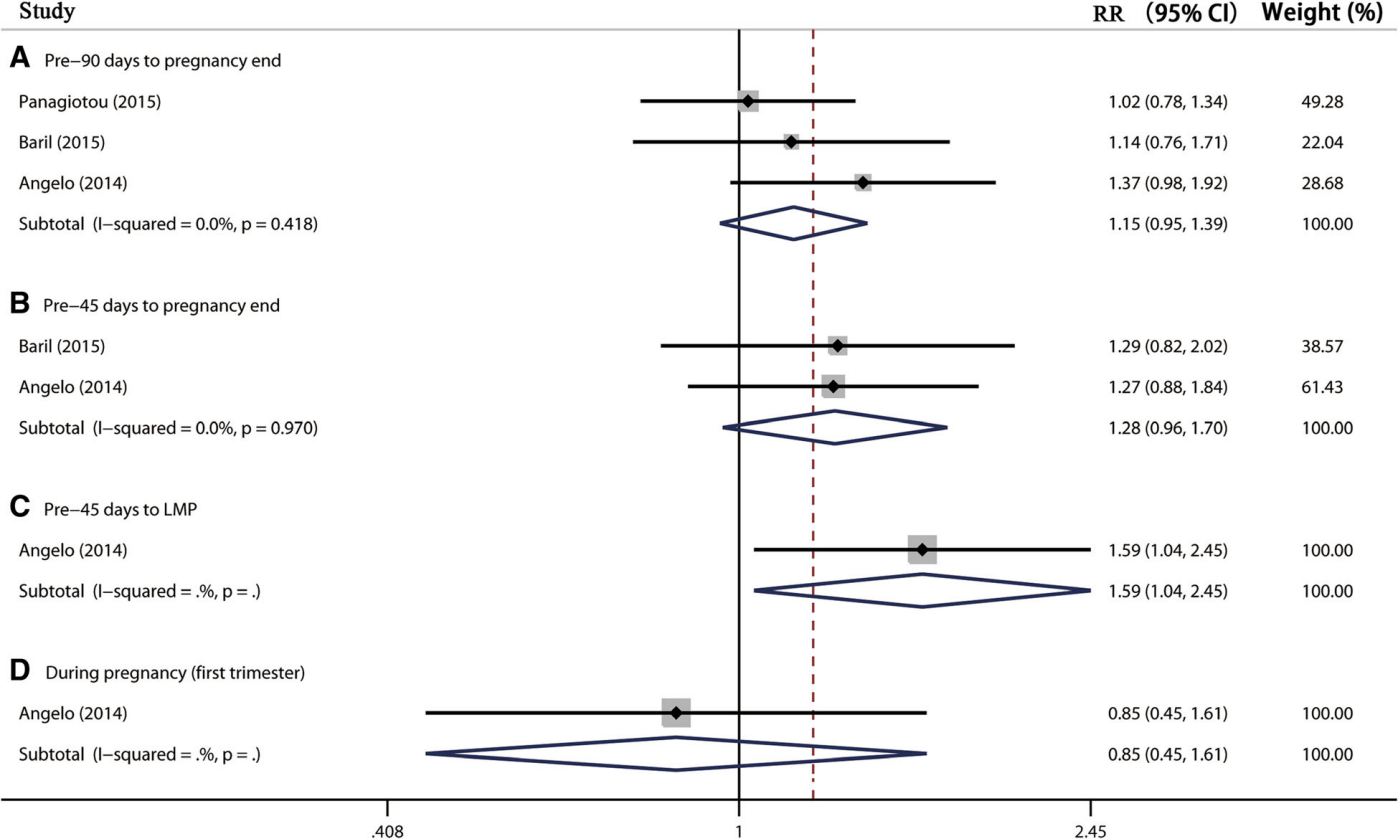
Forest plot of the association between 2vHPV vaccination and spontaneous abortion. RR, relative risk; LMP, last menstrual period

Three studies [14–16], including 5484 participants, reported the association between 2vHPV vaccination and spontaneous abortion. The results showed 2vHPV vaccination during Pre-90 days to pregnancy end and Pre-45 days to pregnancy end, seem to increase the risk of spontaneous abortion, but without statistical significance (pooled RR 1.15, 95% CI: 0.95–1.39, *I*^*2*^ = 0.0% and pooled RR 1.28, 95% CI: 0.96–1.70, *I*^*2*^ = 0.0%) (Table 2, Fig. 3). However, 2vHPV vaccination during pregnancy was not associated with spontaneous abortion (pooled RR 0.85, 95% CI: 0.45–1.61), respectively (Table 2, Fig. 3). The pooled RDs of spontaneous abortion of these three exposure windows were 1.6% (95% CI: − 0.8-4.1%, *I*^*2*^ = 0.0%), 2.7% (95% CI: − 0.7-6.1%, *I*^*2*^ = 0.0%), and − 2.0% (95% CI, − 10.7-6.6%, *I*^*2*^ = 0.0%), respectively (Table 2). However, the result showed that 2vHPV vaccination during Pre-45 days to LMP, seemed to increase the risk of spontaneous abortion with RR of 1.59 (95% CI: 1.04–2.45) and RD of 5.6% (95% CI: 0.2–11.1%) (Table 2). Sensitivity analysis by using alternative effect measures and statistical models did not show significant changes both in pooled results. Subgroup analysis showed the pooled RRs of adjusted and unadjusted results were 1.03 (95% CI: 0.90–1.19, *I*^*2*^ = 0.0%) and 1.37 (95% CI: 0.98–1.39) in Pre-90 days to pregnancy end exposure window, respectively (Table 3).

**Table 2:** Association between exposure to HPV Vaccination and spontaneous abortion

Vaccine	Exposure windows	Number of studies	Vaccine exposure group (total, n)	Vaccine exposure group (SA, n)	Control group (total, n)	Control group (SA, n)	RR	95% CI	*I* ^*2*^	RD (%)	95% CI (%)	*I* ^*2*^
2vHPV	Pre-90 days to pregnancy end	3	1176	155	4308	521	1.15	0.95–1.39	0.0	1.6	−0.8-4.1	0.0
	Pre-45 days to pregnancy end	2	680	85	1046	99	1.28	0.96–1.70	0.0	2.7	−0.7-6.1	0.0
	Pre-45 days to LMP	1	317	48	316	30	1.59	1.04–2.45	–	5.6	0.2–11.1	–
	During pregnancy (0–12 GWs)	1	137	16	124	17	0.85	0.45–1.61	–	−2.0	−10.7-6.6	–
4vHPV	Pre-45 days to pregnancy end	4	2557	238	4257	389	0.88	0.73–1.06	0.0	−1.3	−2.9-0.3	0.0
	Pre-45 days to LMP	3	1199	141	2405	276	1.00	0.80–1.24	0.0	0.1	−2.5-2.7	0.0
	During pregnancy	2	1358	97	2771	209	0.79	0.62–1.01	0.0	−1.8	−3.5-0.1	0.0
9vHPV	Pre-30 days to first 30 days of pregnancy	1	97	17	1418	122	2.04	1.28–3.24	–	8.9	1.2–16.6	–

*2vHPV* bivalent HPV vaccine, *4vHPV* quadrivalent HPV vaccine, *9vHPV* nonavalent HPV vaccine, *LMP* Last menstrual period, *SA* Spontaneous abortion, *RR* Relative risk, *RD* Risk difference

**Table 3:** Subgroup analysis of association between exposure to HPV Vaccination and spontaneous abortion

	Number of studies included	Vaccine exposure group (total, n)	Vaccine exposure group (SA, n)	Control group (total, n)	Control group (SA, n)	RR	95% CI	*I* ^*2*^	RD (%)	95% CI (%)	*I* ^*2*^
2vHPV (Pre-90 days to pregnancy end)											
Data source											
Clinical Trials base	2	846	121	3676	464	1.16	0.88–1.55	42.7	2.0	−1.7-5.7	43.5
Database	1	330	34	632	57	1.14	0.76–1.71	–	1.3	−2.7-5.3	–
Adjusted data or not											
Adjusted ^a^	2	711	84	3859	471	1.03	0.90–1.19	0.0	–	–	–
Unadjusted	1	465	71	449	50	1.37	0.98–1.39	–	–	–	–
4vHPV (Pre-45 days to pregnancy end)											
Data source											
Clinical trials base	2	213	31	1486	180	0.85	0.57–1.27	0.0	−2.2	−7.8-3.4	0.0
Database	2	2344	207	2771	209	0.89	0.72–1.09	20.2	−1.0	−2.7-0.7	0.0
Adjusted data or not											
Adjusted ^a^	2	2344	207	2771	209	1.06	0.85–1.32	13.7	–	–	–
Unadjusted	2	213	31	1486	180	0.85	0.57–1.27	0.0	–	–	–

*a* adjusted by age at conception, *2vHPV* bivalent HPV vaccine, *4vHPV* quadrivalent HPV vaccine, *LMP* Last menstrual period, *SA* Spontaneous abortion, *RR* Relative risk, *RD* Risk difference

### Association between 4vHPV vaccination and spontaneous abortion

**Fig. 4:**
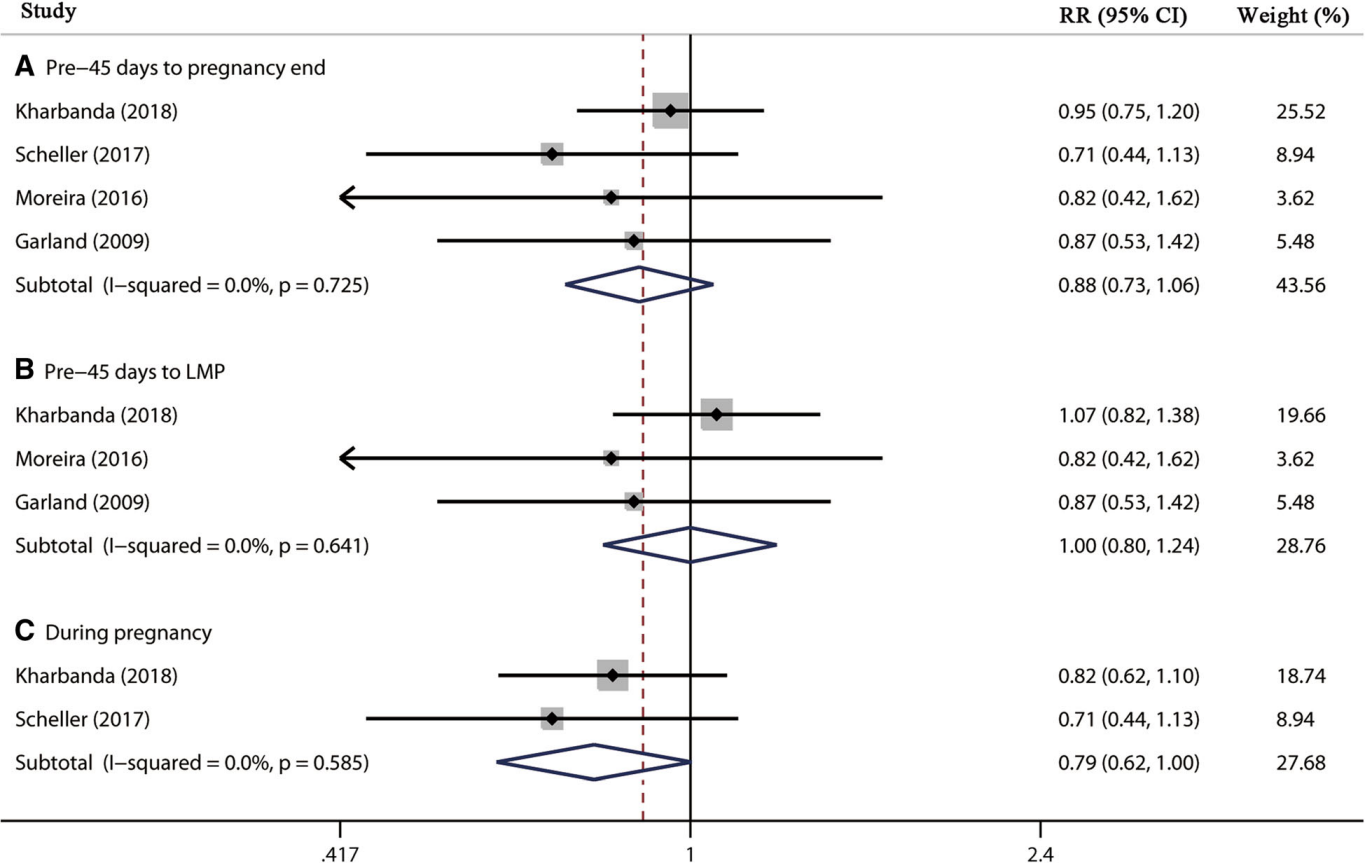
Forest plot of the association between 4vHPV vaccination and spontaneous abortion. RR, relative risk; LMP, last menstrual period

Four studies [2, 12, 13, 18], including 6814 participants, reported the association between 4vHPV vaccination and spontaneous abortion. The results suggested that 4vHPV vaccination during Pre-45 days to pregnancy end, Pre-45 days to LMP, and during pregnancy exposure window did not increase the risk of spontaneous abortion with pooled RR of 0.88 (95% CI, 0.73–1.06, *I*^*2*^ = 0.0%), 1.00 (95% CI, 0.80–1.24, *I*^*2*^ = 0.0%), and 0.79 (95% CI, 0.62–1.01, *I*^*2*^ = 0.0%), respectively (Table 2, Fig. 4). The pooled RDs of spontaneous abortion of these three exposure windows were − 1.3% (95% CI, − 2.9-0.3%, *I*^*2*^ = 0.0%), 0.1% (95% CI, − 2.5-2.7%, *I*^*2*^ = 0.0%), and − 1.8% (95% CI, − 3.5-0.1%, *I*^*2*^ = 0.0%), respectively (Table 2). Sensitivity analysis using alternative effect measures and statistical models did not show significant changes both in pooled results of these three exposure windows.

Subgroup analysis showed both the data of databases or clinical trials based did not indicate 4vHPV vaccination increased the risk of spontaneous abortion in Pre-45 days to pregnancy end with pooled RR of 0.89 (95% CI, 0.72–1.09, *I*^*2*^ = 20.2%) and 0.85 (95% CI: 0.57–1.27, *I*^*2*^ = 0.0%), respectively (Table 3). Similarly, the pooled result of adjusted and unadjusted RR was 1.06 (95% CI, 0.85–1.32, *I*^*2*^ = 13.7%) and 0.85 (95% CI, 0.57–1.27, *I*^*2*^ = 0.0%) in Pre-45 days to pregnancy end exposure window, respectively (Table 3).

### Association between 9vHPV vaccination and spontaneous abortion

Only one study reported the association between 9vHPV vaccination and spontaneous abortion [13]. In the study, the exposure window of 9vHPV vaccination was within 30 days before conception and within the first 30 days of pregnancy. The RR of 9vHPV vaccination for spontaneous abortion was 2.04 (95% CI, 1.28–3.24), and the RD was 8.9% (95% CI, 1.2–16.6%) (Table 2).

## Discussion

To the best of our knowledge, this is the first meta-analysis to evaluate the risk of peri-conception and pregnancy exposure of HPV vaccination on spontaneous abortion. Our results suggested that administration of 2vHPV during Pre-90 days to pregnancy end, Pre-45 days to pregnancy end, and during pregnancy, did not increase the risk of spontaneous abortion with pooled RRs of 1.15 (95% CI, 0.95–1.39), 1.28 (95% CI, 0.96–1.70), and 0.85 (95% CI, 0.45–1.61), respectively. However, during the exposure window of Pre-45 days to LMP, 2vHPV vaccination seemed to increase the risk of spontaneous abortion with RR of 1.59 (95% CI, 1.04–2.45). In addition, our results showed 4vHPV vaccination during Pre-45 days to pregnancy end, Pre-45 days to LMP and during pregnancy, were not associated with higher risk of spontaneous abortion with pooled RR of 0.88 (95% CI, 0.73–1.06), 1.00 (95% CI, 0.80–1.24), and 0.79 (95% CI, 0.62–1.01), respectively. For 9vHPV, although only one study included, considering the high risk (RR = 2.04, 95% CI: 1.28–3.24), the possibility that administration of 9vHPV during peri-conception and pregnancy may increase spontaneous abortion cannot be ruled out.

Although a substantial number of clinical trials on the safety of HPV vaccines have been completed worldwide, the evidence of the association between peri-conceptional or pregnancy exposure of 2vHPV vaccination and spontaneous abortion was limited. In a long term follow-up study for pregnancy outcomes in women enrolled in the Costa Rica HPV Vaccine Trial [14], the authors showed a higher risk of spontaneous abortion at 13 to 20 weeks for HPV vaccination (RR 1.35, 95% CI: 1.02 to 1.77). In another pooled analysis of clinical trials study, the incidence of spontaneous abortion among women who conceived during less than 90 days from 2vHPV vaccination was higher than that in control group (13.7% vs. 9.2%) [17]. Similarly, in 2014, based on data from 42 completed/ongoing clinical trials, Angelo et al. reported that comparing with control group, 2vHPV vaccination during Pre-45 days of LMP increased the incidence of spontaneous abortion when compared with the control group (RR 1.59, 95% CI: 1.04–2.45) [16]. Since routine pregnancy testing before HPV vaccination was not advised in currently clinical practice in the guidelines of WHO, America and many other countries [8, 26, 27], and unintended pregnancies account for a large proportion (estimated rate up to 40%) of pregnancies [28], the number of women in the world who inadvertent administration of HPV vaccine during peri-conceptional period or during pregnancy was enormous. If peri-conceptional or pregnancy exposure of HPV vaccine increases the risk of miscarriage, even if the risk is very weak, we should be vigilant.

For 4vHPV, no evidence suggested that administration during peri-conception and pregnancy might increase the risk of spontaneous abortion. Based on national database of Denmark, the risk of 4vHPV vaccination during pregnancy for spontaneous abortion in different gestational period was investigated in detail [29]. In fully adjusted model, the authors indicated that 4vHPV vaccination during pregnancy did not increase the risk of spontaneous abortion during any of the gestational periods (< 7 weeks, 7 weeks, 8 weeks, 9 weeks, 10 weeks, 11 weeks, 12 weeks, and 13–22 weeks). In addition, different vaccine doses administered before and during pregnancy (1 dose or > 2 doses) were also not associated with higher risk of spontaneous abortion. Yet unexpectedly, the adjusted results showed that 4vHPV vaccination during pregnancy was even associated with lower spontaneous abortion rate during gestational week of 7, 9 and 11 [29].

In 2018, Moreira et al. reported the safety profile of 9vHPV by combined analyzed 7 phase III clinical trials [13]. The authors indicated that 9vHPV vaccine was well tolerated in subjects aged 9 to 26 years with any adverse events (AE) profile similar to that of the 4vHPV vaccine. However, the incidence of spontaneous abortion in conception date within 30 days of 9vHPV vaccination group (20.0%, 17/85) was higher than that of 4vHPV vaccination group (9.2%, 8/87) [13]. In addition, when compared with the conception date not within 30 days of 9vHPV vaccination group, the incidence of spontaneous abortion in conception date within 30 days group was significantly higher (17.5%, 17/97 vs. 8.6%, 122/1418) with RR of 2.04 (95% CI, 1.28–3.24) [13]. These results suggested that 9vHPV vaccination during peri-conception and early pregnancy may increase the risk of spontaneous abortion. At the same time, given the small number of pregnant women in the vaccination group, the results in this study require further research to confirm.

Based on the results of published studies, the risk of administration of HPV vaccine in different exposure windows for spontaneous abortion seemed different. Wacholder et al. reported the incidence of spontaneous abortion of conception during 0–30 days, 31–60 days, and 61–90 days between the nearest 2vHPV vaccination was 15.6% (24/153),14.5% (18/124), and 13.6% (16/117), respectively [17]. Angelo et al. showed the incidence of spontaneous abortion of pregnant women who vaccinated 2vHPV within 45 days before pregnancy, and during the first trimester was 15.1% (48/317) and 11.7% (16/137), respectively [16]. Whether the incidences of spontaneous abortion after exposure of HPV vaccine in different periods were similar or not, and whether exposure of HPV vaccine in a specific period around conception probably increased the risk of spontaneous abortion need additional research.

Although the association between HPV vaccination and spontaneous abortion has aroused great interest in recent years, the potential mechanism is rare known. One explanation is that spontaneous abortion may be caused by repeated antigen exposure [15, 30]. Another explanation is that the ASO4 adjuvant in vaccine may alter the maternal immune system during early pregnancy, and then increase the risk of spontaneous abortion [14, 31]. However, these two potential mechanisms are controversial, and need further exploration [12, 14].

The main strength of this study was firstly conducting the meta-analysis, which targeting an unanswered important clinical question, the association between the unintended exposure of HPV vaccination at the peri-conceptional period or during pregnancy and spontaneous abortion. Meanwhile, the present meta-analysis involved two principal limitations which should be addressed. Firstly, exposure time windows of vaccination in included studies were not unified. Although the association between HPV vaccination and spontaneous abortion in long exposure time windows (e.g. Pre-90 days to pregnancy end) was showed in this study, for specific exposure time periods such as during 90 to 30 days before LMP, the risk of HPV vaccination was also unclear. Four exposure windows were generalized in this study, and this categorization may strengthen the effect of vaccination exposure farther from pregnancy, while in turn reduce the effect of exposure closer to pregnancy. Secondly, since only four included studies (two studies for 2vHPV and 4vHPV each) reported the adjusted RR of HPV vaccination for spontaneous abortion, we were unable to assess the contribution of confounding factors in our results. Women vaccinated during the peri-conceptional period were more likely unintended pregnancy, or women vaccinated during pregnancy were more likely unaware of pregnancy. When compared with awareness of pregnancy or planned pregnancy, all these women are more likely to be exposed to risk factors for spontaneous abortion, such as consumption of cigarettes or alcohol. Consequently, an individual patient data meta-analysis was needed to confirm the association between HPV vaccination in the peri-conceptional period or during pregnancy and spontaneous abortion.

## Conclusion

Evidence of the association between peri-conceptional or pregnancy exposure of HPV vaccination and spontaneous abortion was limited, however, a real association cannot totally be ruled out. Additional research is warranted to assess the impact of exposure of 2vHPV or 9vHPV vaccination on spontaneous abortion. Considering the insufficient evidence, women at childbearing age should preferably avoid unintended HPV vaccination during pregnancy.

## Additional file

Additional file 1: Search strategies. (DOCX 18 kb)

## Data Availability

The datasets used and/or analysed during the current study are available from the corresponding author on reasonable request.

## Abbreviations

2vHPV: Bivalent human papillomavirus vaccine
4vHPV: Quadrivalent human papillomavirus vaccine
95% CI: 95% confidence interval
9vHPV: Nonavalent human papillomavirus vaccine
CENTRAL: Cochrane Central Register of Controlled Trials
HPV: Human papillomavirus
LMP: Last menstrual period
NOS: Newcastle–Ottawa Scale
OR: Odds ratio
PRISMA: Preferred Reporting Items for Systematic Reviews and Meta-Analyses
RD: Risk difference
RR: Relative risk
