# Supplementary material for: Peri-conceptional or pregnancy exposure of HPV vaccination and the risk of spontaneous abortion: a systematic review and meta-analysis
Source: BMC Pregnancy Childbirth. 2019 Aug 19;19:302. doi: 10.1186/s12884-019-2425-1 (PMC6699066; doi:10.1186/s12884-019-2425-1)
Supplement: Supplementary file 1 — Search strategies. (DOCX 18 kb) [file 12884_2019_2425_MOESM1_ESM.docx]

**Additional file 1 Search strategies**

**一. P****ubmed (Search date: April 9, 2019)**

# 1 Pregnant Women[mh] OR Pregnancy[mh] OR Pregnan*[tiab] OR conception[tiab] OR gravid*[tiab] OR gestation*[tiab] OR Childbearing[tiab] OR Child bearing[tiab] OR parturient[tiab] OR parturition[tiab]

# 2 Papilloma[mh] OR Papillomaviridae[mh:noexp] OR Alphapapillomavirus[mh] OR Papilloma*[tiab] OR Alphapapillomavirus*[tiab] OR HPV[tiab]

# 3 observation*[tiab] OR cross section*[tiab] OR cross-section*[tiab] OR case-control[tiab] OR case control[tiab] OR cohort*[tiab] OR follow-up[tiab] OR follow up[tiab] OR longitudinal[tiab] OR prospective[tiab] OR retrospective[tiab] OR clinical trial*[tiab] OR Controlled Trial*[tiab] OR RCT[tiab] OR database*[tiab] OR regist*[tiab] OR surveillance[tiab]

# 4 (#1 and #2 and #3)

**二. Embase (Ovid) (Search date: April 9, 2019)**

# 1 exp named groups by pregnancy/ or exp pregnancy/ or (Pregnan* or conception or gravid* or gestation* or Childbearing or Child bearing or parturient or parturition).ab,kw,ti.

# 2 papilloma/ or papillomaviridae/ or exp alphapapillomavirus/ OR (Papilloma* or Alphapapillomavirus* or HPV).ab,kw,ti.

# 3 (observation* or cross section* or cross-section* or case-control or case control or cohort* or follow-up or follow up or longitudinal or prospective or retrospective or clinical trial* or Controlled Trial* or RCT or database* or regist* or surveillance).ab,kw,ti.

# 4 (#1 and #2 and #3)

**三. Cochrane Central Register of Controlled Trials (CENTRAL) (Ovid) (Search date: April 9, 2019)**

#1 MeSH descriptor: [Pregnant Women] explode all trees

#2 MeSH descriptor: [Pregnancy] explode all trees

#3 Pregnan*:ti,ab,kw or conception:ti,ab,kw or gravid*:ti,ab,kw or gestation*:ti,ab,kw or Childbearing:ti,ab,kw or Child bearing:ti,ab,kw or parturient:ti,ab,kw or parturition:ti,ab,kw

#4 (#1 or #2 or #3)

#5 MeSH descriptor: [Papilloma] explode all trees

#6 MeSH descriptor: [Papillomaviridae] explode all trees

#7 MeSH descriptor: [Alphapapillomavirus] explode all trees

#8 Papilloma*:ti,ab,kw or Alphapapillomavirus*:ti,ab,kw or HPV:ti,ab,kw

#9 (#5 or #6 or #7 or #8)

#10 observation*:ti,ab,kw or cross section*:ti,ab,kw or cross-section*:ti,ab,kw or case-control:ti,ab,kw or case control:ti,ab,kw or cohort*:ti,ab,kw or follow-up:ti,ab,kw or follow up:ti,ab,kw or longitudinal:ti,ab,kw or prospective:ti,ab,kw or retrospective:ti,ab,kw or clinical trial*:ti,ab,kw or Controlled Trial*:ti,ab,kw or RCT:ti,ab,kw

#11 (#4 and #9 and #10)
